# More than what we eat: Investigating an alternative pathway for intact starch granules in dental calculus using Experimental Archaeology

**DOI:** 10.1016/j.quaint.2022.03.004

**Published:** 2023-04-20

**Authors:** Sarah Delaney, Michelle Alexander, Anita Radini

**Affiliations:** a BioArCh, Department of Archaeology, The University of York, Wentworth Way, York, UK; b York JEOL Nanocentre, The University of York, Science Park, York, UK

**Keywords:** Ancient starch, Dental calculus, Food processing, Medieval archaeology

## Abstract

Starch granules and other plant tissues are commonly found as part of the microdebris assemblage analysed within dental calculus. These are often interpreted as evidence of past diets. However, many of the starch granules extracted from dental calculus are intact, and do not show evidence of alterations as a result of being processed for consumption. This research examines if plant material can accidently enter the mouth while being processed for a meal, with a focus on starch granules. Grinding experiments were performed on three types of cereal grains (wheat, oat and millet). We compare the presence of intact and altered starch granules in mouthwash samples (in place of dental calculus samples) from individuals involved in grinding and also from samples in the environment surrounding the grinding activity. This experiment is a proof of concept aimed to expand experimental research in the field of dental calculus analysis and to encourage the exploration of pathways beyond direct and deliberate consumption.

## 1 Introduction

Starch granules are one of the most ubiquitous typologies of microremains retrieved during the analysis of ancient human dental calculus, a deposit on teeth formed by the mineralisation of dental plaque (also known as tartar). Starch granules entrapped within human dental calculus can provide valuable information on past diet (e.g. Henry et al., 2011; Jovanović et al., 2021; Mariotti Lippi et al., 2017; Mickleburgh and Pagán-Jiménez, 2012; Wesolowski et al., 2010; Zhang et al., 2017), particularly where plant macroremains are not well preserved, e.g. deep in the human past (Hardy et al., 2012, 2016; Radini et al., 2016b; Copeland and Hardy, 2018). Starch granules found in dental calculus are usually thought to be direct evidence of plants being consumed deliberately for dietary purposes (Copeland and Hardy, 2018). By comparison, archaeobotanical remains from archaeological contexts such as soil (Hart, 2011; Haslam, 2009; Lucarini et al., 2016) and artefacts (e.g.stone tools) (Field et al., 2009; Lucarini et al., 2016; García-Granero, 2020; Hamon et al., 2021; Zanina et al., 2021) provide indirect evidence of plant remains in the diet. Dental calculus is also more ubiquitous than other forms of direct evidence for diet such as coprolites and gut samples (Leonard et al., 2015). Additionally, starch granules themselves tend to be exceptionally well preserved in the calculus matrix (Jovanović et al., 2021; Hardy et al., 2016a, 2016b; Radini et al., 2017). The archaeological integrity of microremains in archaeological dental calculus is widely accepted, provided that the correct sample decontamination procedures are followed (Cristiani et al., 2016; Radini et al., 2017; Warinner et al., 2014).

Dental calculus has received growing attention by scholars in recent years and studies have shown that a great variety of information related to the consumption of starchy food can be retrieved from human dental calculus in the form of starch granules. These microremains improve the archaeological visibility of important economic plants and staple foods in different periods of time and geographic locations, ranging, for example from rice (*Oryza* spp.) and millets (Panicoideae, possibly *Setaria italica* and *Panicum miliaceum*) in Bronze age samples from Shilinggang, southwestern China, to pulses (Fabaceae) and cereals (Triticeae) from medieval Leicester, England (Radini et al., 2016a; Zhang et al., 2017). Starch from tubers have also been found, for example, revealing the consumption of sweet potato (*Ipomoea batatas*) in Rapa Nui, Easter Island, dating prior to European contact (Tromp and Dudgeon, 2015).

As pointed out by several scholars, earlier research on microremains analysis of dental calculus focused mainly on plant remains and deliberate consumption of starchy food (Henry and Piperno, 2008; Hardy et al., 2009, 2012; Henry et al., 2011, 2012), however, in the past few years, research has begun to explore other pathways for the inclusion of a wide variety of microremains in dental calculus. These include particles (such as pollen, animal fibres, pigments and even cotton) which can derive from the surrounding environment, and specific crafting activities, showing dental calculus to be potentially a novel source of evidence regarding living conditions of past populations (e.g. Hardy et al., 2016a, Hardy et al., 2016b; Juhola et al., 2019; Radini et al., 2016a, 2019). This new evidence is growing in importance, and it is indicating that there is a need to better understand the pathways or various ways in which different typologies of remains become incorporated in the dental calculus matrix. In this paper, we use an experimental methodology to examine a possible pathway of inclusion for intact starch granules in dental calculus beyond that of diet: accidental inhalation/ingestion by oral breathing during food processing by grinding stone tools. Our experimental methodology is carried out using methods and quantities that are realistic in terms of activities carried out as part of everyday life, in this case the use of querns and typical meal-sized quantities of grain. This hopefully provides us with some indication of the likelihood of inhaling these particles through the mouth when carrying out this activity as a typical daily task. Previous studies have shown that experimental work on inclusion of particles in saliva during activities can be used as a ‘rapid testing of hypothesis’ for exploring pathways of inclusion of microremains in dental calculus (Radini et al., 2019).

## 2 Research framework and aims of the paper

### 2.1 Research framework

**Fig. 1:**
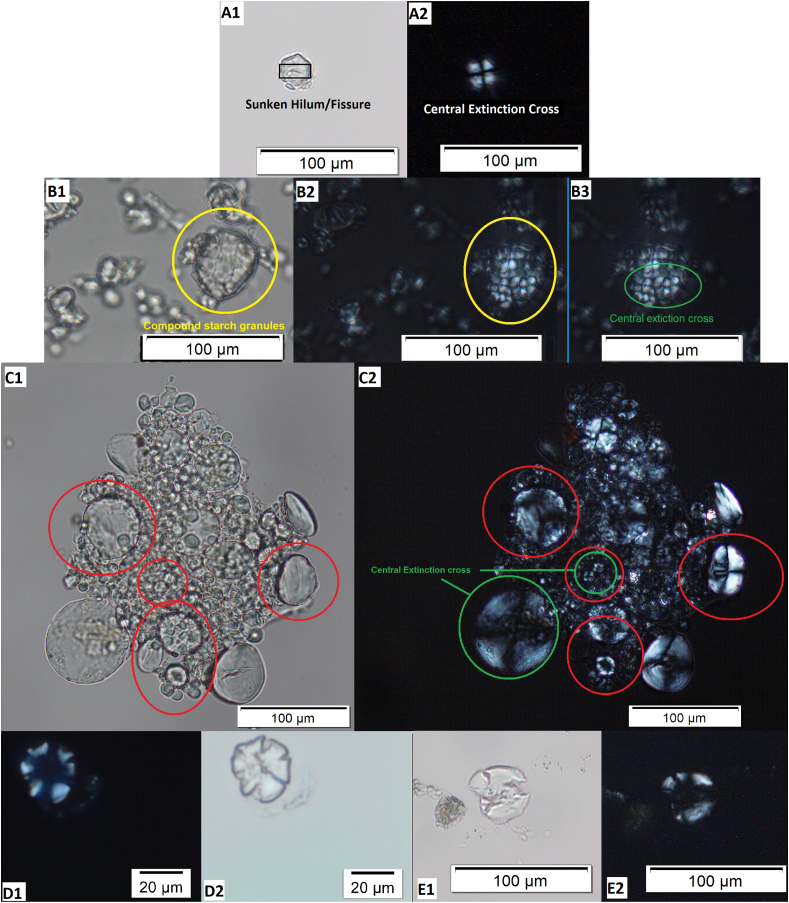
**A***: Panicum miliaceum* (millet) starch granules showing diagnostic features. A1, Morphology: small polyhedral single grains - hilum centrally located with sunken fissures (shown in in black rectangle). A2, Extinction cross (visible under polarised light. **B**: *Avena sativa* (oat) starch granules showing diagnostic features. B1, Morphology: very small polyhedral compound grains circled in yellow. B2, Extinction cross (visible under polarised light central and right and circled in green) central clear X shape. **C**: *Triticum durum* (durum wheat) starch granules showing diagnostic features. C1, Morphology: round to oval shape with bimodal size (below 20 μm and above μm) circled in red - hilum centrally located. C2, Clear extinction cross (visible under polarised light and circled in green). **D1-2**: Altered starch granule from dental calculus from Littlemore Priory, England (pictures by Sarah Delaney), altered possibly due to grinding or other processing. **E1-2**: Starch granule altered due to grinding. Found in an environmental sample that was placed 50 cm away from the saddle quern while the quern was being used to grind wheat. For interpretation of the references to colour in this figure legend, the reader is referred to the Web version of this article.

Starch granules are organic, semi-crystalline structures made up of two types of glucose chains, amylopectin and amylose (Henry et al., 2009) and are effectively the ‘energy storing molecules ‘for plants, lodged in a special organelle known as amyloplasts (Leonard et al., 2015). The production of starch granules is influenced by a number of varying environmental factors, and genetics (Raviele, 2011). Starch granules can be very rarely diagnostic to genus or species level, and their identification requires clear visibility of certain diagnostic features such as the hilum and extinction cross, their size, as well as various other morphological features (Fig. 1, A-C). However, their identification to tribe and family level can be achieved in archaeological contexts in a more reliable way if the granules are well preserved. For further information, Barton and Torrence (2015) provides an expanded overview on starch analysis in archaeology.

Processing of starchy food can be beneficial for consumption and in some cases is necessary, for example, by making food more easily digestible and/or more palatable, and even safer to eat (Hardy et al., 2015; Liener, 1962). Processing methods, such as prolonged grinding or cooking techniques, have been experimentally shown to alter starch granules (Chantran and Cagnato, 2021; Henry et al., 2009; Mickleburgh and Pagán-Jiménez, 2012). The impact of food processing on different grains is relatively well studied for both archaeological purposes and within food sciences (Chantran and Cagnato, 2021; Gomez et al., 1992; Henry et al., 2009; Saul et al., 2012; Waterschoot et al., 2016). Cracking and gelatinisation/swelling are two common types of alterations that are associated with food processing for consumption and have, as a result, been used to identify this activity in archaeological populations. Gelatinisation occurs when starch granules are heated typically above 60 °C, causing them to lose their molecular organisation and diagnostic features. Exposure to moisture and heat cause swelling and alterations of the shape of the starch granules, which leads to loss of birefringence and reduction in the definition of the extinction cross (Copeland and Hardy, 2018; Hardy et al., 2009; Mariotti Lippi et al., 2017). More relevant to this study is the ‘cracking’ on starch granules which occurs as the result of the plant being exposed to dry heat and/or grinding (Buckley et al., 2014). Cracking alters the shape of the starch granule (Fig. 1, D-E), often flattening it, and can cause a reduction in birefringence. These alterations to starch granules frequently make identifying a starch granule to even family level difficult, due to the loss of diagnostic features.

The identification of starch granules found in dental calculus often relies on their features being intact. While intact starch granules are a relatively common find in dental calculus, the supposed link between their presence and the direct consumption of plant foods does not take into account that starchy foods are likely to have undergone some form of processing before consumption. Copeland and Hardy (2018) noted that starch granules in dental calculus should, in theory, only be representative of food that has not been sufficiently cooked or is raw, which would allow the starch granules to be intact and diagnostic, and without evidence of alterations, including those potentially caused by salivary amylase. The presence of intact starch granules in calculus, however, needs to be better understood. Radini et al. (2016a, 2016b) hypothesised that some of the starch granules from the cereals present in dental calculus samples from medieval Leicester may have accidently entered the mouth during milling the grain or handling flour. Experimental studies that are aimed at better understanding the pathways of inclusion of microremains in dental calculus are needed to improve our interpretations, but these are currently limited in number. Work by Henry et al. (2009), demonstrated that starch granules could still be assigned to tribe level even when altered. Saul et al. (2012) explored the survival of starch granules in experimental carbonisation experiments using ceramic vessels, proving a range of alterations occurred in starch granules, depending on the heat applied over time, including the repetitive use of the vessel for cooking. Dozier's (2016) experimental work on cereal grinding clearly showed that starch granules are easily airborne and can be dispersed some distance away from processing sites. However, surprisingly, fewer starch granules were generally found in indoor samples, and while some were found more than 20 cm away, they were in relatively low quantities. The above confirms the presence of intact starch granules in the environment caused by grinding does occur, which implies that it is possible to inhale intact starch granules as the result of cereal processing.

While this paper focuses on the processing of starchy food, it is worth mentioning the experimental work conducted by Radini et al. (2019) exploring the accidental ingestion of dust particles, in that instance generated by the grinding of the semi-precious stone lapis lazuli during pigment preparation. That work proved that a flux of particles inside the human mouth was generated during the breakdown of lithological material and suggested that many other ‘dusty’ crafts can equally leave evidence in ancient dental calculus, if dust generated during the processing of raw material enters the human mouth. Such studies demonstrate it is important to understand how different human activities may have produced airborne by-products sufficiently fine to enter the human mouth and the saliva, and become incorporated in the dental calculus matrix. Starchy food processing may be one of these processes.

### 2.2 Aims

The studies and observations previously discussed clearly show the importance of ‘realistic experimental settings’ for assessing starch survival and alteration during food preparation and consumption. This was an important consideration in the design of our experiment, which asks: is it possible for intact starch granules to be accidently ingested via oral breathing as the result of human activity occurring in the environment, such as food processing, specifically cereal grinding? Does the grinding method or type of grain being ground have an impact on the type of starches that may be inhaled? This paper presents an experiment that aims to answer these questions and improve our understanding of pathways to inclusion beyond deliberate consumption. We examine whether grinding cereal grains using quern stones results in the presence of starch granules in the saliva and assess their state of preservation when the quantity of grain and processing tools are similar to those used by people in the past.

## 3 Materials and methods

### 3.1 Selection of grains and grinding equipment

Three types of cereal grain were ground, millet (*Panicum miliaceum*, tribe Paniceae), wheat *(Triticum durum*, tribe Triticeae) and common oat (*Avena sativa,* tribe Aveneae). These cereals were selected for several reasons. Firstly, the starch granules produced by these crops are morphologically and diagnostically different: wheat starch is composed of single oval to round starch granules (large type A and small type B), with a bimodal distribution, and are easily contrasted in modern reference material with those from oat (compound starch granules) and those in millet (single starch granules). This enabled the counts of starch granules produced during each experiment to be as accurate as possible. Secondly, using these different grains enabled us to assess if the starch morphology of crops influences their airborne properties. For example, starch granules from oats are known as compound granules because they tend to be in composite particles made up of multiple granules, which might lead to them being less airborne than single starch granules of wheat. Thirdly, these crops played a significant role in the diet of human societies in the past. For example, wheat and oat were dietary staples in many parts of Medieval Europe (Stone, 2006). Other starchy plants, such as tubers, roots and legumes, were omitted as they were processed in numerous different ways other than grinding.

**Fig. 2:**
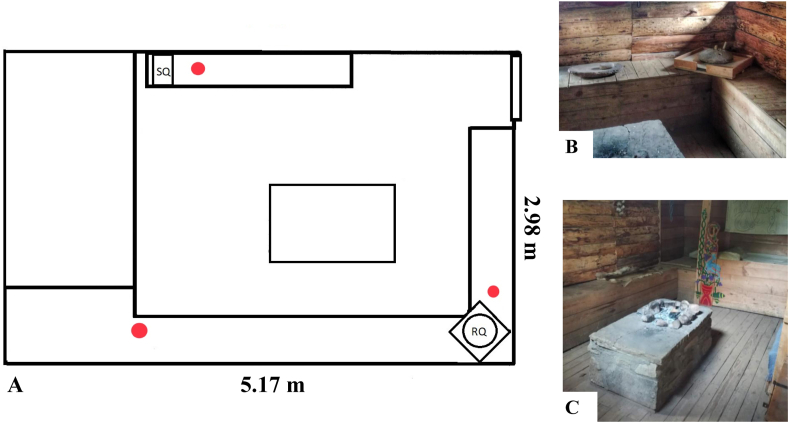
**A**: Plan of Viking Style House the area used for the experiment. SQ indicates location of Saddle Quern and RQ indicates Rotary Querns. Red circles note the location of environmental samples (Plan drawn with adobe illustrator based on hand drawn plan made on day of experiment). **B** and **C**: photos of the area at Murton Park, North Yorkshire, where the experiment took place. (For interpretation of the references to colour in this figure legend, the reader is referred to the Web version of this article.)

The main experiment (i.e., grinding grain) took place at a ‘Viking’ domestic house structure at Murton Park, Museum of Farming, Yorkshire, UK in September 2019. Photographs and a plan of the house are provided in Fig. 2. The house had one doorway that remained open, but all window openings were shuttered for the duration of the experiment (no glass was present).

**Fig. 3:**
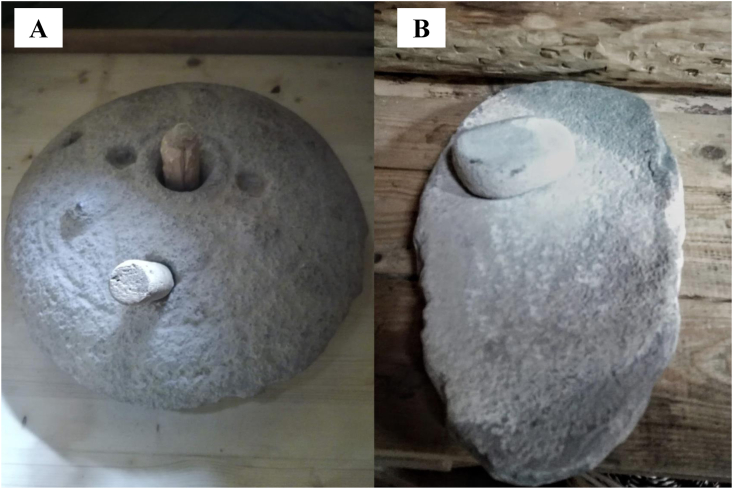
Querns used during the experiments. **A**: Rotary quern; **B**: saddle quern used for the experiment.

Two different types of querns were used: a saddle quern and a rotary quern (Fig. 3). These represent two different grinding methods that were commonly used in the past. The saddle quern involves exposed grinding (referred to as ‘open’ for the purposes of this paper) - the grain is placed in the centre of a large stone and then ground with a smaller stone that is held in the hand. The rotary quern is a more closed (referred to as ‘closed’) system made up of two almost hemispherical-shaped large, worked stones comprising a top cover and bottom base. The cover stone has a wooden handle used to rotate the stone creating the pressure and motion needed to grind the grain against the bottom base. Whether these grinding techniques involved open or closed grinding surfaces is important, as it may affect how particles are dispersed in the environment, and thus how easily particles may be inhaled. Additionally, these querns use different motion and pressure in the grinding which may also have an impact. Part of the experiment aimed to assess whether these two methods resulted in a measurable difference in the total quantity of debris inhaled, or, the quantity of intact versus altered starch granules that may be inhaled.

While previous experiments on the effect of cooking or food processing tended to use only two to three grains/seeds (Capparelli et al., 2011; Crowther, 2012; Henry et al., 2009), we opted for a larger quantity of grain that could more realistically represent one meal or a loaf of bread, c. 250g per grain for each quern.

### 3.2 Environmental samples

Environmental samples were taken to compare with the starch granule content of the mouthwash samples, recording the presence of both intact and altered starch granules. They enabled us to examine how starch granules travelled within the space, and whether granules from species ground earlier in the experiment remained in the environment.

### 3.3 Experimental protocol

All procedures outlined below were carried out in accordance with the Ethical Approval for Research on Human Tissues approved by the Biology Ethics Committee, University of York (reference ARA201907). Multiple samples were taken in order to examine the presence of intact and altered starch granules in the mouth and the environment. As the activity was conducted in a facility where tools may be in close proximity with the public, a careful cleaning protocol for the querns was adopted.

Control samples for microscopic analysis were taken from the querns during the first phase of cleaning, prior to experiments. This was to give an indication of any microremains, and in particular starch granules, that might have been present from their storage location. Samples taken both from the saddle quern and rotary quern prior to the start of the grinding experiment included plant remains, such as starch granules (many of which may possibly be *Avena* sp.), plant epidermal tissues and non-plant remains. The rotary quern control included mineral particles (including quartz), most of which probably derived from the quern stone and also examples of pollen and insect hairs. It was not possible to determine when these debris were deposited, and so this material could derive from the stones’ original/early use, or their storage conditions. The querns were then very carefully cleaned to avoid cross contamination. As the experiment aims to assess accidently inhaled airborne starch granules, it must be stressed that:

1. Any residual material dislodged from the querns during processing would be insignificant in amount compared to the considerable quantity of fresh starch present as result of the experiment.
2. Even if there is a evidence of a small amount of residual material being inhaled, this would still be evidence of inhalation during food processing.

The flour produced from each phase of the experiment was initially removed by hand, followed by a bristle brush, a soft toothbrush, and finally compressed air was used to remove any particles that may have entered the cracks and crevices of the grinding equipment. Samples of the flour were also taken for microscopic analysis. This procedure was repeated after the processing of each type of grain and at the end of the experiment. Our experiments were recorded by the museum curators as part of the ‘biography’ of those tools for future reference.

Environmental samples from inside of the house where the grinding took place were taken using three dust traps in the form of sterile petri dishes, 90 mm in diameter. Their placement locations give an indication of the quantity of starch granules in the immediate environment of the querns throughout the experiment and allowed us to assess the ‘rain’ dispersal of starch granules at a lower level than the mouths of the individuals. Two dust traps were placed 50 cm away from each quern, and the third was placed 3.2m from the rotary quern, in the corner of the room between the two querns (Fig. 2, A). New traps were put out for each grinding session. Each petri dish was sealed after each session to avoid contamination. There were a total of 18 environmental samples (i.e. three for each of the six grinding sessions).

**Fig. 4:**
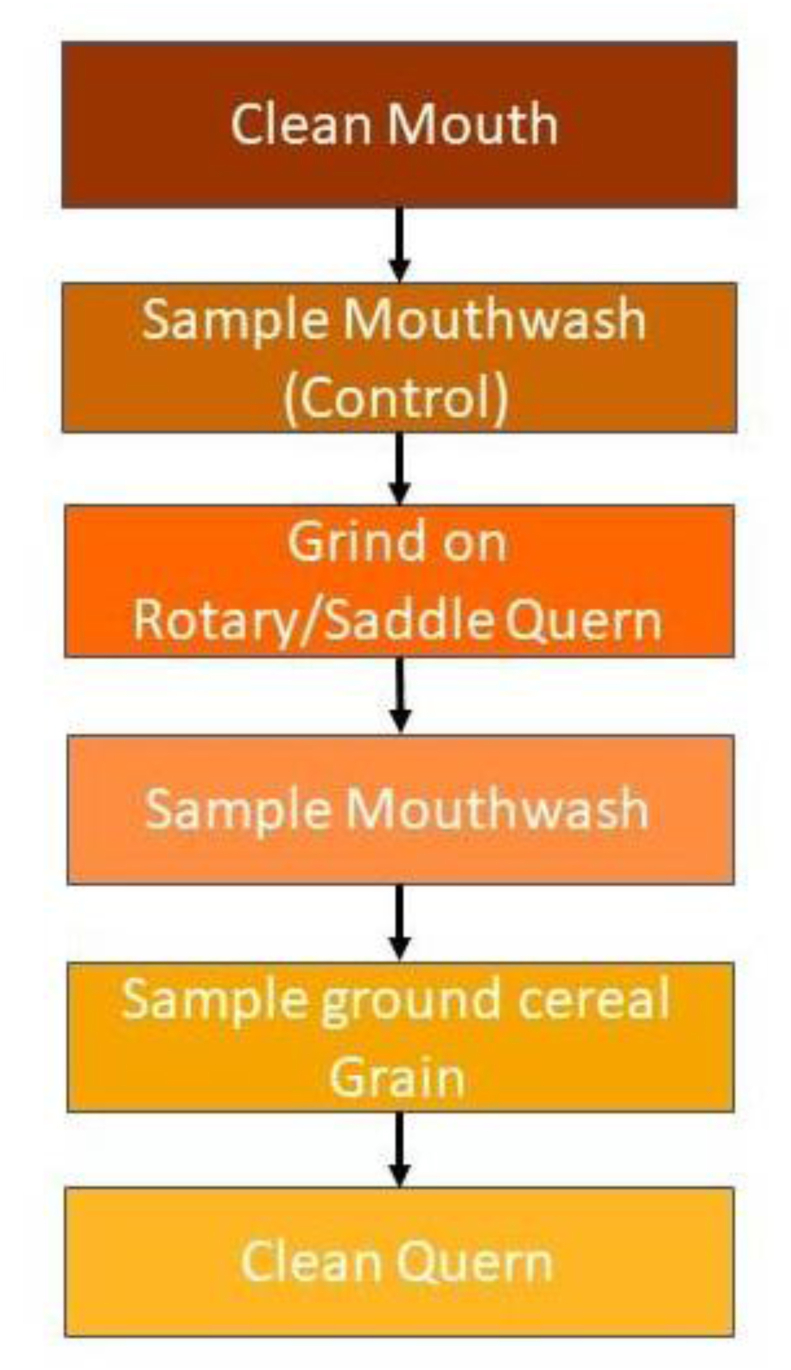
Flowchart showing steps and process for experiment (beginning with cleaning the mouth and control samples, followed by grinding, sampling and then cleaning the querns).

Our sampling protocol follows the one successfully used by Radini et al. (2019) adapted to the task of grinding small meal sized amounts of cereals. A protocol flowchart is provided in Fig. 4. Prior to beginning the grinding process for each grain, individuals participating in the experiment cleaned their mouth thoroughly by rinsing with distilled water, brushing their teeth and flossing before rinsing their mouth again to remove as much debris as possible. A control mouthwash sample of 1.5 ml was taken from each individual after their mouth had been cleaned. We used this to determine any background debris that may have been present in the mouth despite cleaning. The mouth was also cleaned between grains using the same procedure. The time taken to grind each batch of grain varied depending on the quern, the individual and the grain. After each grain was ground, mouthwash samples (using 1.5 ml of ultrapure water) were taken, which were directly deposited from the mouth into 1.5 ml Eppendorf tubes. The times are noted in Table 1. After each grain had been ground, samples of the ground grain (flour) were also taken for comparison with the particles that were inhaled.

**Table 1:** Time spent on each experiment by each individual.

Grain	Quern	Individual 1 (minutes)	Individual 2 (minutes)
**Wheat**	**Rotary**	20	25
	**Saddle**	30	45
**Oat**	**Rotary**	15	15
	**Saddle**	10	10
**Millet**	**Rotary**	15	15
	**Saddle**	10	25

### 3.4 Analysis of the samples

After the experiment, the mouthwash samples were stored in a fridge (4 °C) to prevent debris alteration or degradation by preventing the activation of amylase in saliva (De Almeida et al., 2008). Prior to analysis, samples were centrifuged at 3600 RPM to separate the supernatant and pellet. The samples were analysed within 72 h of collection.

Slides for microscopic analysis were prepared in a clean laboratory where dust and dirt are monitored to reduce the risk of contamination to secure the integrity of the analysis. A 200 μl aliquot of the mouthwash containing the pellet was pipetted and mounted with 50:50 distilled water: glycerol onto a clean microscope slide, which was then immediately covered with a glass slide cover and sealed with clear nail varnish.

Slides were analysed using an Olympus Microscope BX53M, under 200x magnification to gain an overview of the amount of debris. Slides were then examined under 400x magnification with all starch granules recorded and counted in a spreadsheet with the necessary photographs taken. Starch granules were classified as ‘intact’, ‘altered’, ‘other species’ and ‘nondiagnostic’. When needed, the magnification was increased to 630x. Observations were completed by scanning the samples with cross -polarised light, which allowed for birefringent material to be made visible and observe any alteration of the extinction cross that could be linked to damage by processing. Counting was not carried out for the samples of the ground grain taken from the querns due to time constraints. Any data gained from that analysis would not have added to the interpretations of this study. Samples were disposed of safely after analyses, following the standard method applied by the Archaeology Department for the disposal of biological material and following ethical guidelines.

### 3.5 Presentation of the results

The amount of time spent on each experiment by each individual is provided in Table 1 by grain and quern type. The results for the control samples taken before grinding each grain on each quern can be found in Supplementary Table 1, which includes the number of intact (identified as being diagnostic starch granules coming from each grain) altered, from other species, or nondiagnostic starch granules. Those which say ‘Type a’ or ‘Type b’ refer to the types of Triticeae starch granules recovered.

Below the control samples in Supplementary Table 1, we present the data concerning the starch granules retrieved from the mouth washes. The results from mouthwash samples post-grinding using each quern and grain show the number of intact (identified as being diagnostic starch granules deriving from each grain) altered, from other species, or nondiagnostic starch granules. The diagnostic starch granules from oat (*Avena sativa*) were counted per diagnostic compound starch granule (unless otherwise stated) as the compound nature of oat is diagnostic, unlike with other starch granules from the other grains which were counted per starch granule due to their non-compound nature.

## 4 Results

Starch granules and microdebris identified from environmental and mouthwash samples are described by category below.

### 4.1 Starch granule morphology from ground grain (flour) from the querns post grinding

**Fig. 5:**
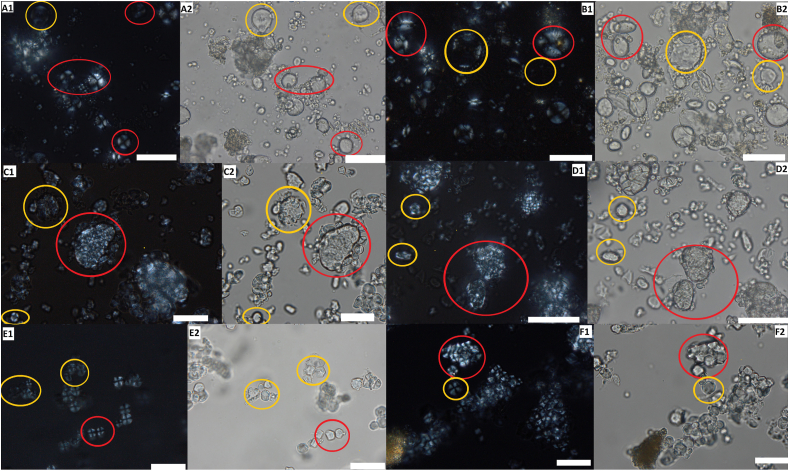
Examples of starch granules from flour ground using the querns during this experiment. Scale bars are 50 μm. Wheat on Rotary (A) and saddle (B) quern; oat on rotary (C) and saddle (D) quern; millet on rotary (C) and saddle (D) quern. Starch granules circled in red are intact and those circled in yellow are altered. Pictures numbered 1 were taken under cross-polarised light, those numbered 2 were taken without cross-polarised light. (For interpretation of the references to colour in this figure legend, the reader is referred to the Web version of this article.)

Starch granules in the flour produced during grinding were found to be present as a mixture of intact and damaged granules (examples in Fig. 5A–F). There were varying forms of damage, such as cracking, shape alteration and altered/reduced birefringence, which were present to varying degrees. Some of the ground starch granules from oat appear to be compound and intact, while others are separated and show damage (Fig. 5, C-D). Starch granules both with alterations and without evidence of alterations were present in all the ground grain samples, showing that both types of starch granules may be present in ground grain after this type of processing, (the counts for these are provided in the SI). These observations are consistent with other experimental work on this subject, where grinding has been shown to cause cracks, alter the shape and alter and reduce birefringence (Buckley et al., 2014).

### 4.2 Starch granules in the environment

**Fig. 6:**
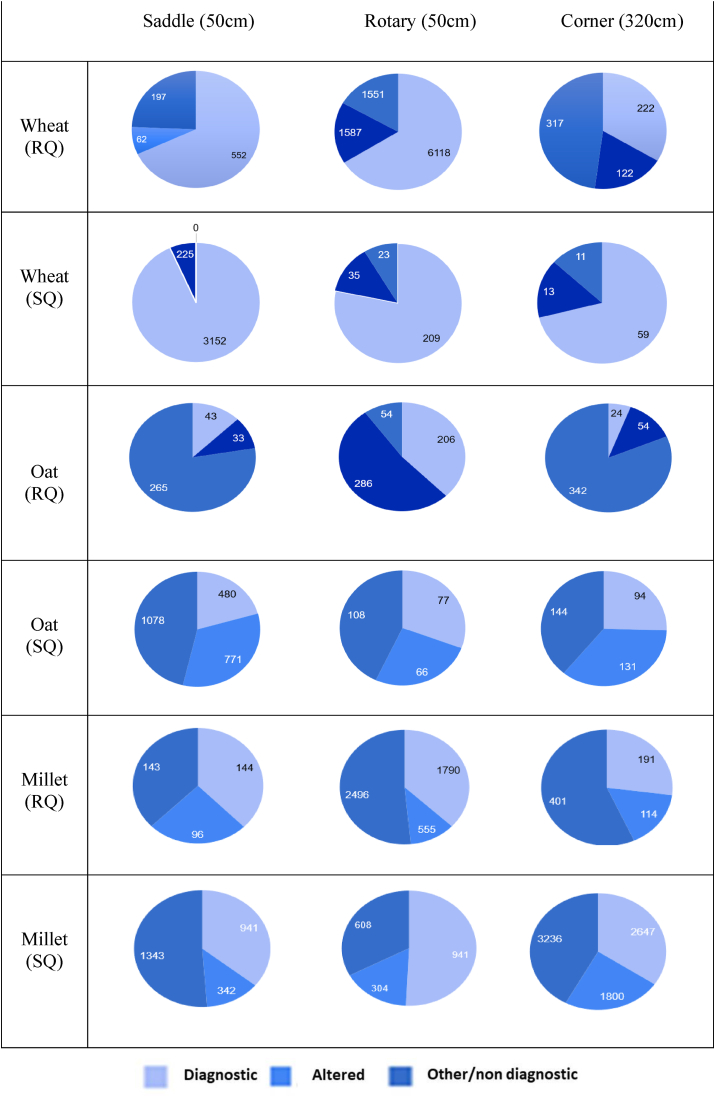
Pie Charts showing the results from environmental samples taken during the experiment, showing the number of intact ground grain (identified as being diagnostic starch granules coming from each grain) altered, from other species, or nondiagnostic starch granules. The diagnostic starch granules from oat were counted per diagnostic compound starch granule as the compound nature of oat is diagnostic, unlike with other starch granules from the other grains which were counted per starch granule due to their non-compound nature. SQ: Saddle Quern/RQ: Rotary Quern. Saddle (50 cm) was 50 cm from the saddle quern. Rotary (50 cm) was 50 cm from rotary quern. Corner (320 cm) was in the corner of the room which was 320 cm from both querns.

Counts and typologies of starch granules found in dust traps where grinding took place are presented in Fig. 6. The highest number of starch granules (of the species being ground, altered and other species/non-diagnostic starch granules) were found in traps positioned closest to each quern that was being used at the time the samples were taken. Starch granules were present in all samples, however, counts were quite variable, for example, ranging between 222 and 6118 for intact wheat starch granules ground on the rotary quern. Altered starch granules were also present in all samples and were again most numerous in the samples collected closest to the querns in use at the time of sampling. In four of the oat samples, the number of altered starches was higher than identifiable and intact starches. This may be due to the fact that oat starch granules can only be positively identified when in compound form rather than as single granules. Altogether, these results indicate that the act of grinding leads to airborne particles around the physical space in which grinding takes place, and that these particles include both intact and altered starch granules.

### 4.3 Starch granules from mouthwash samples

**Fig. 7:**
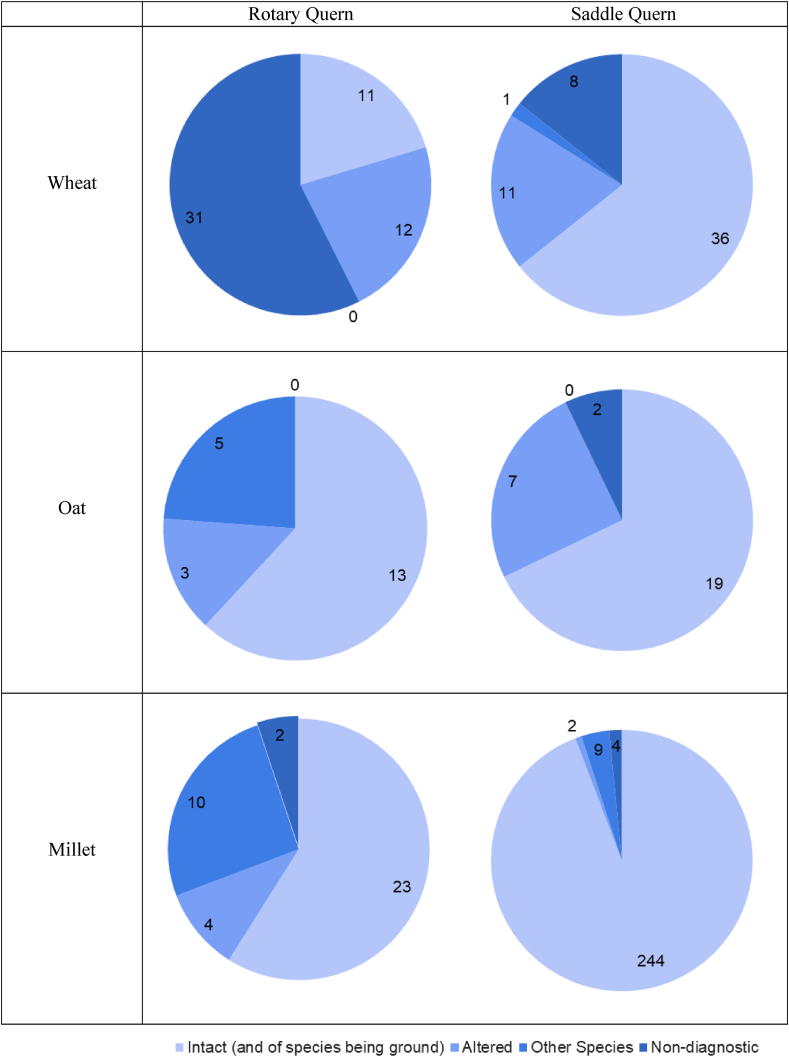
Pie chart showing the results for starch granules present in mouth wash samples after each grain ground on saddle and rotary quern, results are the total for Individual 1 and Individual 2 added together. It includes the diagnostic starch granules for the species being ground, altered starch granules, other species identified, and non-diagnostic.

**Fig. 8:**
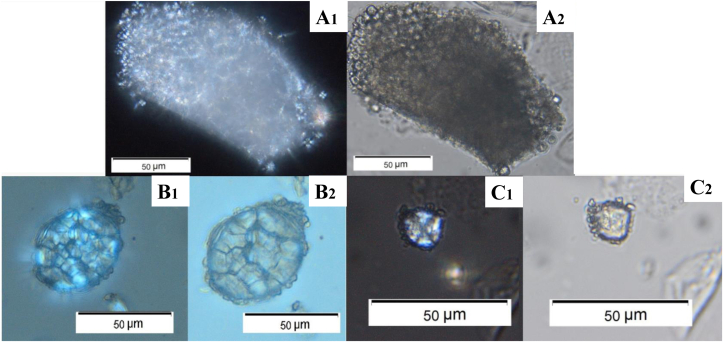
Intact Starch Granules from mouthwash samples. Pictures numbered 1 were taken under cross-polarised light, those numbered 2 were taken without. **A**: from saddle quern wheat inhaled by Individual 1. **B**: from saddle quern oat inhaled by Individual 2. **C:** from rotary quern millet inhaled by Individual 1.

**Fig. 9:**
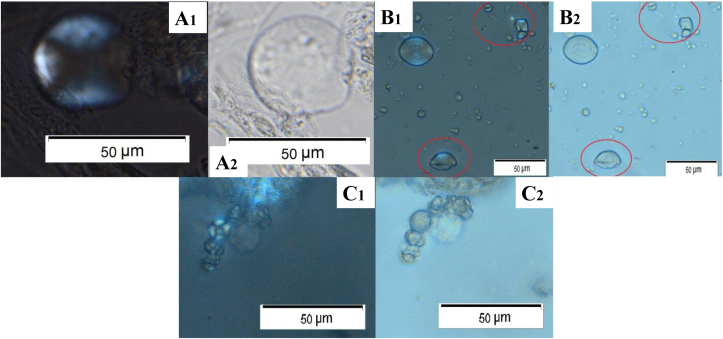
Altered starch granule from mouthwash samples. Pictures numbered 1 were taken under cross-polarised light, those numbered 2 were taken without. **A**: enlarged and flattened millet starch from saddle quern, inhaled by Individual 1. **B**: damaged wheat starch from saddle quern (circled in red) inhaled by Individual 2. **C**: oat starch, no longer in its complete and intact compound form, from saddle quern, inhaled by Individual 2. (For interpretation of the references to colour in this figure legend, the reader is referred to the Web version of this article.)

All data related to starch granule counts and identifications from mouthwash taken as control prior to grinding and those immediately after grinding can be found in Supplementary Table 1, which shows the starch granule counts for each mouthwash sample. The results are also plotted as pie charts in Fig. 7, with each chart showing the combined count of both Individual 1 and 2 for that session, rather than showing the counts for each individual separately, for clarity. Images of intact starch granules identified in mouthwashes taken after grinding are presented in Fig. 8 and examples of alterations on starch are visible in Fig. 9. The starch granules in Fig. 8 still show birefringence, and do not show evidence of cracking or folding. Additionally, in Fig. 8a, the starch granules still appear to be partially present in the amyloplast, showing it was not entirely damaged during grinding. In Fig. 8b, the oat starch granules are still present in compound form, rather than being broken apart as a result of the grinding process. In contrast, in Fig. 9, starch granules show shape alteration, varying reduction in birefringence, and folding. The latter is especially visible in Fig. 9b. As the data provided in Fig. 7 and Supplementary Table 1 shows, the majority of starch granules were recovered in the samples immediately after grinding, and there was very little found in the control samples prior to grinding or after the mouth had been cleaned following grinding.

The first control samples taken before the first grinding session (wheat on the rotary quern) had no starch granules present. The control samples taken prior to each grinding session thereafter had zero to six individual starch granules present in them, and these were never from the species that were to be ground during that session. The starch granules that were present in all other control samples were consistently possible wheat starch granules (i.e. A type and B type) and altered starch granules. Therefore, despite strict cleaning of teeth and mouth, some starch granules remained in the mouth, but in very low numbers. Three possible hypotheses are plausible:

1. Some starch granules were residual in the environment from the previous grinding activity, remained airborne and were accidently inhaled.
2. Inhaled starch granules could reach further into the respiratory tract and then be expelled out into the mouth again (such as through breathing), the respiratory tract therefore may act as a reservoir for starch granules inhaled, increasing the chances of them to remain in the mouth for longer.
3. A combination of both 1 and 2.

The results from the samples taken after grinding indicate that starch granules, both intact and altered, are inhaled via oral breathing during the grinding process. Only one mouthwash sample taken by one individual after grinding did not contain both intact and altered starch granules (Individual 1, oat on the rotary quern).

The number of starch granules inhaled varied widely between the different querns, cereals and Individuals. Samples taken by Individual 2 consistently had a higher number of total starch granules present in each mouthwash sample compared to Individual 1. The most dramatic example for this is the case of millet ground on the saddle quern where the total number of millet granules identified for Individual 1 was three, whereas for Individual 2, the total was 248. For Individual 1, five out of six mouthwash samples had a higher number of altered starch granules identified than those from the species that was being ground. For Individual 2, however, intact starch granules from the species being ground were consistently found to be present at a higher rate than altered starch granules. The pie charts reflect this higher number of starch granules from ground species, especially as Individual 2 had consistently a higher number of identified starch granules in their mouthwash samples.

Oat is present in fewer numbers compared to both wheat and millet, potentially indicating that the compound starch granules are less airborne than the single granules, and/or the small granules from broken oat compounds may be more subjected to salivary clearance. It is possible that as oat was counted in its compound form, this reduced the number of starch granules counted. However, these mouthwash samples also contained a generally low number of starch granules that were altered, derived from other species or nondiagnostic.

## 5 Discussion

### 5.1 General overview

Our experiment resulted in five key findings:

1. Uncooked starch granules enter the human mouth during the grinding of all the grain types using quern stones. Some of this starch was also dispersed in the environment.
2. The quantity of debris generated was not large, and the number of starch granules that entered the mouth were limited for each experiment, but they could accumulate if no mouthwash was undertaken.
3. Many of the starch granules retrieved in the experiment were found to be intact.
4. The number of starch granules that entered the mouth varied between species.
5. Differences in the number of starch granules found in the salivary samples of the two researchers were different.

### 5.2 Starch granule inhalation and cereal processing

It has long been known that processing of large quantities of grains produces dust that can impact human health (e.g. Duke, 1935). Our experiment shows that the processing of ‘household meal-sized’ quantities of grains (Stone, 2006, pp 20–22), as would have been common in the recent past, releases starch granules, some of which are intact, into the environment and can be inhaled through oral breathing and become incorporated into saliva. This key result suggests that intact starch granules retrieved in calculus could be the result of this pathway of inclusion. It has been noted that some other garden produce, such as legumes would be processed at home too (Woolgar, 2006); it would be therefore interesting to repeat this experiment with legumes and other foods that tend to be more moist than cereal grains, and, as a result, potentially produce less airborne dust.

While the counts for diagnostic starch granules might appear to be quite low, certain factors need to be considered. Firstly, starch granules do not tend to be found in high counts in dental calculus samples compared to both the number present in plants themselves and the amount of plant foods an individual consumes in their lifetime (Copeland and Hardy, 2018). It should also be remembered that grinding cereals would have been a frequent occurrence in the past, potentially a daily activity (Berman, 2007; O'Sullivan and Nicholl, 2011). Debris from grinding grain could also remain present in the environment for an unknown period of time, and therefore the potential for debris to enter the oral cavity and become entrapped in dental calculus would increase over time. During the experiment, we carried out several mouthwashes, both for pre-grinding controls and post-grinding of each grain species. The frequent mouthwashes may have altered the processes of salivary clearance, as well as reduced the concentration of starch granules that remained in the saliva over time. We processed a relatively small quantity of grain (representing a single small meal); whereas a larger quantity and prolonged grinding will increase the amount of starch granules both in air, settled dust and mouth. Additionally, our mouthwashes may not have retrieved all starch granules in the mouth, and this is a potential limitation.

A greater number of intact starch granules were generally found across both the mouthwash and environmental samples. This finding could indicate that altered starch granules are not as easily dispersed as intact granules, and intact granules tend to be dispersed quickly before more extended processing leads to them being cracked or broken. The results are unclear as to whether using a saddle quern (more exposed grinding) or a rotary quern (which, while covered had grains at varying stages of being ground emerging from the side) has an impact on the nature of the debris that is inhaled.

Our results also indicate that there is much variation between individuals in terms of what and how much is inhaled via oral breathing, which would have an impact on how much debris has the potential to be trapped in dental calculus. This is due to a number of factors including whether someone is breathing through their mouth (for example Individual 1 is not a mouth breather, whereas Individual 2 is), how much they are talking, how often someone breathes out through their mouth (as this would expel saliva and particulates from the mouth), as well as salivary clearance rate.

There are also other non-human factors that may impact on debris being inhaled, such as the physical characteristics of the grain and processing time. As to be expected, starch granules from grain that was in the process of being ground were more likely to be inhaled. However, the results from the environmental and mouthwash samples show that starch granules from wheat are more likely to remain in the environment and continue to be inhaled after grinding. This might be due to wheat taking much longer to be ground (between 20 and 45 min for both querns), thereby extending the time period for these starches to be released in the environment. This experiment would need to be repeated and extended to examine these factors further. Such work is currently in progress for non-dietary remains funded by the Wellcome Trust (Radini, grant number 209869/Z/17/Z). Finally, The presence of starch granules in the environmental dust traps shows that a portion of the starch granules freed from the grains during grinding can reach the ground during processing. In many studies on stone tools, soil samples are often investigated for the presence of starch granules that can cause contamination; in such studies that presence of starch in soil is considered a potential agent of contamination of modern origin. This work suggests that some of such starch could actually be generated by the use of the stone tools and grinding implements, and therefore may not be strictly indicative of a contamination.

### 5.3 Raw, processed or inhaled

The results of the experiment show that deliberate consumption is not the only pathway for intact and altered starch granules to enter the mouth and become entrapped in dental calculus. This is especially notable for intact starch granules, considering most of the current published experiments show that starch granules become altered when processed for consumption. Additionally, the ground grains (flour) sampled from the quern also showed that starch can remain intact after processing, further indicating that starch granules can survive grinding. This, in combination with mouthwash samples and environmental samples, shows that unaltered starch granules may be released into the environment, and while some may be quickly inhaled, much may remain.

Our findings also show that the amount of starch granules retained in the mouth during grinding is very limited compared to the quantity of starch that would flood the oral cavity during eating. Additionally, it is possible that intact starch granules have a better chance to survive in the saliva and in the dental calculus matrix when compared to damaged starch, despite bacterial activity in the human mouth. Starch granule accumulation in the environment as a result of plant processing has been noted by Dozier (2016), therefore representing a potential pathway of inclusion by transfer, meaning that starch granules may also be ingested due accumulating on items in the surrounding environment, and as a result may be accidentally inhaled or ingested at later point. This could suggest that if they land on the face, lips (see Radini et al., 2019), bowls, eating/cooking utensils etc. then they can become incorporated with food or simply the object could be put in the mouth. This is another pathway that is difficult to account for. Overall, it is likely that not all starch granules found in dental calculus will be the result of food processing, but this pathway should be taken into consideration.

### 5.4 Broader contextualisation of the data within the medieval period

It is important to consider whether different populations from different contexts would show variation. This study fits into broader research that is focused on the medieval period, comparing debris from dental calculus samples from a range of different sites across Medieval England, particularly between rural and urban contexts. In samples from both types of context in the overarching project, both intact and altered starch granules from cereals were found (for example see Fig. 1 D). The medieval period is therefore considered here as an example of how social and cultural practice may affect exposure to airborne starch granules.

Grinding cereal for e.g. beer, bread and pottage, was a common activity during the medieval period which would have taken place both inside and outside the home, exposure varying by occupation and gender. These were both a dietary staple in Medieval England, consumed on a daily basis (Pearson, 1997; Stone, 2006; Woolgar, 2006). Due to the importance of bread in the diet in particular, there were strict laws regarding the making and selling of this product, which came into practice during the 13th century and was widely enforced throughout England (Davis, 2004).

In terms of variation by gender and occupation, in urban areas, bakers were commonly men, whereas women were typically the majority in the occupations associated with brewing and retailing of ale, especially in the later medieval period (Bennett, 1987, pp. 120–125; Mate, 1999, pp. 39). Both of these occupations would have led to greater exposure to inhaling debris from grain, perhaps more for milling than brewing, although there is evidence that some brewhouses housed hand mills or querns for grinding malt (Dyer, 2013). Given the gender norms regarding these industries, there may be differences present between particular individuals identified as male and female from urban settlements in the medieval period that may be related to these occupations.

In the rural context, however, grinding grain was considered a domestic task carried out by women, possibly daily. From around 1150 to 1400 CE, most villages or manors had a mill controlled by the manorial lord where people were to bring their grain to be ground (Hanawalt, 1986; Hilton, 2003; Smith, 2009; Bartels, 2012). However, there is evidence this did not always occur. Archaeological and documentary evidence from rural sites suggests that grinding took place indoors and in the home. At Wharram Percy, for example, stone querns were found in domestic contexts and documentary records indicate that there were periods of several years where the mill was not functional when women would have carried out grinding at home (Smith, 2009). According to Bennett (1987, pp. 126–127), prior to the Black Death, women in rural areas would sometimes add to a household income by carrying out milling, baking and brewing, which would likely take place in the area of the household. However, unlike in urban areas, this would not necessarily be treated as a main occupation and could be carried out sporadically (Bennett, 1987, pp. 124; Mate, 1999, pp. 46). As noted by Dyer (2013), however, having grain ground at a mill would save household labour, so if there was access to a mill and people could afford it, they may have been more likely to use it. Overall, it would seem that while both urban and rural populations consumed bread on a daily basis, populations at rural sites would have experienced more exposure to debris from grinding grain in the household than urban contexts, either from direct exposure from grinding or from debris remaining from this activity in the household environment.

A final point that we would like to address is in relation to the scale of food processing. The quantity of starch granules inhaled in this small-scale experiment shows that a low portion of starch granules is accidently inhaled by oral breathing. Individuals with certain occupations (miller, baker and, to a lesser extent, brewers depending on if they processed their own grain) in both rural and urban areas, would have experienced more intense prolonged exposure and presumably will have trapped a greater quantity of such debris in their dental calculus. Large scale production of flour in the past and today in many societies are known to have had a serious effect on human and animal respiratory health (Stobnicka and Górny, 2015), and further experimental work with a larger quantity of grain/flour or mill flour production would be an important future step for this research, and would potentially provide important data on the impact of plant processing on human health in past populations.

### 5.5 Comparison with non-dietary experiments

Even though these experiments were carried out on a different typology of raw material, as well as different quantity and time of grinding in comparison to the lapis lazuli study by Radini et al. (2019), some general observations can be made by comparison. The quantity of starch granules overall after nearly a day of grinding grain, were rather small, compared with the quantity of particulates found in the lapisl lazuli experiment. This can be explained by grains having a certain degree of moisture that lithological materials, such as lapis lazuli, do not. The fine dry dust is more airborne. Furthermore, the energy applied to grind stones from pigments is more significant than that needed to grind grains, generating overall a larger amount of dust during its impact. This brief comparison is here to highlight that different materials and different human activities will generate different quality and typology of dust, and experiments should be conducted on a case by case basis.

### 5.6 Ethical considerations

Finally, we would like to mention that the handling/use of human tissues, of which saliva is considered one, for research purposes are subject to strict ethical agreements, mainly aimed to protect any health information of the participants and the handling of DNA. However, wider ethical implications may be encountered if experimental work is conducted among groups where ethics agreements may not be as straightforward as it is in the case of this paper. We believe that experimental work conducted in this paper and by Radini et al. (2019), suggests it is possible to gain valuable information on flux of particles in the human mouth and saliva that can facilitate our understanding of pathways of inclusions of microremains in calculus with the use of Experimental Archaeology in realistic/accurately recreated circumstances/settings.

## 6 Summary and conclusion

Our experiment has shown that the presence of intact starch granules in dental calculus can derive from grain processing rather than direct consumption alone. Furthermore, the results indicate that it is possible for both intact/diagnostic and altered starch granules to enter the mouth via oral breathing, during processing and even several hours after being ground.

Due to the Covid-19 pandemic, more research is being conducted on the inhalation of particles from the surrounding environment. These studies show how particles can preserve, travel and survive in environments with regards to health, (for example: Bahl et al., 2020; Bourouiba, 2020; Dancer et al., 2020; Kohanski et al., 2020; Peters et al., 2020; Workman and Bleier, 2020). The persistence of microscopic particles in the environment has been recently reported by research from other fields advising the World Health Organisation (WHO), where particles such as bacteria and viruses, which overlap in size with wheat starch granules (Bar-On et al., 2020; Torrence and Barton, 2006, pp. 40–41), can remain present in the environment and are inhalable hours after an event occurred (Dancer et al., 2020; Peters et al., 2020). Findings from these studies show that over time, the particulate load increases. These studies are important when considering the findings from this pilot study, as they show possible pathways for particle inclusion, first in saliva, and subsequently in the dental calculus matrix. The low quantity of granules detected in the experimental mouthwash samples does, however, indicate that the majority of starch granules found in archaeological dental calculus are more likely to be the result of the consumption of starchy food, where a much larger quantity of intact starch granules would be entering the human mouth than previously thought.

Experiments, like the one reported in this article, are necessary to improve understanding of past lifeways and to make interpretations of dental calculus microremains more accurate. We recommend that researchers look beyond the dietary consumption pathway alone when considering the origin of both intact and altered starch granules as inhalation can occur both through processing or even as a result of being present in an environment where cereal grinding has taken place, and other similar occupational pathways should also be considered when examining other types of debris.

## Author contributions

All authors contributed to the research design, with experimental protocols based upon on-going research by AR. SD and AR conducted the experimental work and laboratory analysis of oral samples. SD conducted the sampling and analysis of the environmental samples. SD with AR and MA conducted final data analysis and data contextualisation. SD, AR and MA wrote the paper.

## Data availability

All data from this study are available in this article.

## Declaration of competing interest

The authors declare that they have no known competing financial interests or personal relationships that could have appeared to influence the work reported in this paper.
